# Optimal design of synthetic circular RNAs

**DOI:** 10.1038/s12276-024-01251-w

**Published:** 2024-06-14

**Authors:** Seo-Won Choi, Jin-Wu Nam

**Affiliations:** 1 Department of Life Science, College of Natural Sciences, Hanyang University, Seoul, 04763 Republic of Korea; 2 Bio-BigData Center, Hanyang Institute of Bioscience and Biotechnology, Hanyang University, Seoul, 04763 Republic of Korea; 3 Research Institute for Convergence of Basic Sciences, Hanyang University, Seoul, 04763 Republic of Korea; 4 Hanyang Institute of Advanced BioConvergence, Hanyang University, Seoul, 04763 Republic of Korea

**Keywords:** RNA splicing, Expression systems, Gene expression

## Abstract

Circular RNAs are an unusual class of single-stranded RNAs whose ends are covalently linked via back-splicing. Due to their versatility, the need to express circular RNAs in vivo and in vitro has increased. Efforts have been made to efficiently and precisely synthesize circular RNAs. However, a review on the optimization of the processes of circular RNA design, synthesis, and delivery is lacking. Our review highlights the multifaceted aspects considered when producing optimal circular RNAs and summarizes the available options for each step of exogenous circular RNA design and synthesis, including circularization strategies. Additionally, this review describes several potential applications of circular RNAs.

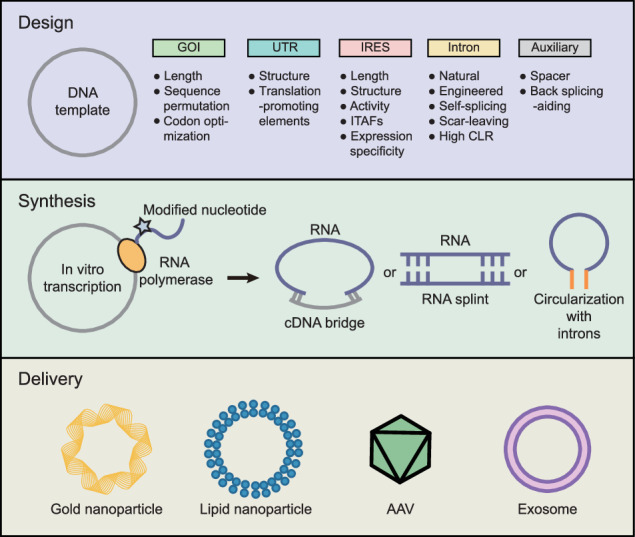

## Streamlining circular RNA synthesis: design, optimization, and delivery strategies

Circular RNAs were once considered errors in splicing. However, we now understand that circRNAs are common and play roles in health and disease, including potential use in creating vaccines. This review examines ways to create and use circRNAs, with a focus on improving their creation and delivery for medical purposes. They discuss various methods, including using natural and synthetic elements to enhance circRNA formation and stability. The main findings suggest that improving the creation and delivery of circRNAs could greatly enhance their medical potential. Researchers conclude that with more development, circRNAs could become powerful tools in gene therapy and vaccine creation, offering new ways to treat diseases. The future implications of this research could transform how we approach disease treatment and vaccine creation, making therapies more effective and long-lasting. This summary was initially drafted using artificial intelligence, then revised and fact-checked by the author.

## Introduction

Circular RNAs (circRNAs) are RNA molecules whose 5′-ends are covalently linked to their 3′-ends, a structure achieved through back-splicing^1–5^. Back-splicing is a process in which a downstream 3′-splice donor is joined to a 5′-splice acceptor that is positioned upstream of the donor. Due to their unique structure, circRNAs were discovered with low expression levels in normal and neoplastic mammalian cells more than three decades ago^1^ but were initially thought to be a byproduct of erroneous splicing. However, later studies revealed that circRNAs are widely expressed not only in mammalian species^2^ but also in a diverse range of other organisms, including other vertebrates^6^, worms^3^, flies^7^, plants^8^, fungi and protists^4^, and lower eukaryotes^5^. High-throughput transcriptome sequencing has revealed that ≥10% of the genes expressed in mammalian cells and tissues can produce circRNAs, further establishing the widespread presence of circRNAs^9^. The biological and clinical importance and potential of circRNAs have been increasingly highlighted in many fields. Numerous studies have reported the expression of thousands of circRNAs under normal and abnormal conditions^10^. CircRNAs are associated with stress-related^11^ and immune-related responses^12^ and have been implicated in many human illnesses, including cancer and neurodegenerative diseases^13,14^.

CircRNAs act as efficient platforms for the expression of functional molecules^15,16^. Especially during the coronavirus disease 2019 pandemic, circRNAs that undergo internal ribosome entry site (IRES)-mediated translation were highlighted as potential mRNA vaccine candidates. Although circRNA-encoded peptides need to be carefully validated^17,18^, it is possible to design and synthesize circRNAs that exhibit robust and stable protein synthesis ability^16,19–21^. Wesselhoeft et al. reported that circRNAs produced 9- and 1.5-fold more proteins than unmodified and modified nucleoside linear RNAs, respectively, with 1.7- to 2.4-fold longer half-lives than linear RNAs in human cells^20^. That same group later demonstrated that nanoparticle delivery and in vivo translation of synthetic circRNAs were feasible^21^. A recent study showed that circRNA vaccines could protect mice^22^ and macaques^15^ against different variants of SARS-CoV-2, with improved efficacy and similar immunogenicity relative to linear RNA vaccines. These promising applications have been noted by industry, with Merck & Co. being one of the important investors. Merck agreed to spend up to $3.75 billion on Orna Therapeutics, Inc., a new startup aiming to develop medicines from synthetic circRNAs^23^. Laronde, another group with a similar goal, had raised $440 million by 2021.

Considering these trends, determining the optimal methods for exogenous circRNA synthesis and delivery, as well as for specific expression in desired tissues, is of utmost importance. In this review, we describe how each step in the expression of exogenous circRNAs can be optimized, along with the strengths and limitations of possible options. Furthermore, we discuss the potential of circRNAs as effector molecules in therapeutic applications.

## Exogenous circRNA synthesis

To express exogenous circRNAs, one should first decide whether to generate circRNAs via in vitro transcription (IVT), circularization, and delivery of the RNA or to inject a DNA construct and generate circRNAs via in vivo transcription. There are various protocols for RNA circularization and in vivo transcription or IVT that lead to different choices for the expression of exogenous circRNAs (Table 1). Here, we describe validated trials for the expression of synthetic circRNAs in vivo and in vitro.

**Table 1:** Strategies for circRNA synthesis.

Type	Backbone	Cell line	Max. efficiency^a^	In vivo/vitro	Reference
Natural introns	circSMARCA5	HEK293T	94%	In vivo	^27^
	circPOLR2A	H9 hESC, HeLa	47%	In vivo	^24^
	HIPK3, ZKSCAN1, EPHB4	HeLa		In vivo	^25^
	laccase2, ZKSCAN1	DL1, SL2, HeLa		In vivo	^26^
	TADA2A-E6 introns	MDA-MB-231, MCF7	50%	In vivo	^28^
	circPVT1/circZKSCAN1	MCF7		In vivo	^34^
	Engineered HIPK3 and ZKSCAN1	HEK293, U87, Huh7		In vivo	^35^
Inverted repeats	CMV promoter + IR + ciRS-7 exons	HEK293		In vivo	^29^
	CamKI introns + Cherry	S2	73%	In vivo	^31^
	pEGFP-C1, CMV promoter + splitGFP + IRES	HEK293		In vivo	^30^
	EGFP intron + ICS	HeLa, HEK293	50%	In vivo	^32^
ECRR	pEGFP-C1, CMV promoter + splitGFP + IRES	HEK293, UT-HeLa, H1299		In vivo	^33^
PIE	*A*nabaena pre-tRNA + spacers + homology arms	HEK293		Both	^20^
	*Tetrahymena* group I intron	–	Comparable to PIE	In vitro	^46^
	*Tetrahymena* group I intron	HEK293T	80%	Both	^47^
	*Clostridium tetani* group II introns	HEK293T	70%	In vitro	^45^
	Engineered T4 td	–		In vitro	^42^
	Permuted T4 td	HEK293T	90%	Both	^44^
Ribozyme	Pol3 promoter + ribozyme + ligation sequence	HeLa, HEK293, HepG2	100%	Both	^36^

No information was available for the empty cells.

*ECRR* engineering of circRNA regulator, *PIE* permuted intron–exon, *IR* inversed repeats, *EGFP* enhanced green fluorescent protein.

^a^Information was retrieved from the original paper or was provided by the authors.

### Generation of circRNAs by in vivo transcription

**Fig. 1:**
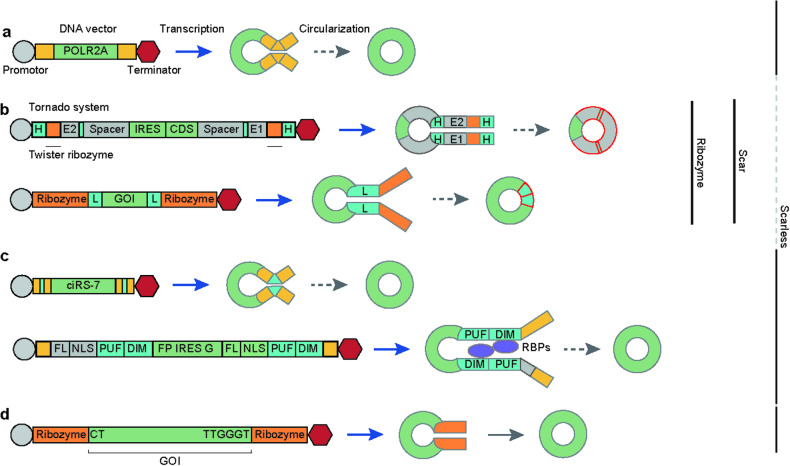
Types of endogenous and synthetic circRNA backbones. **a** Endogenous circularization methods that have been used or mimicked. **b** A highly efficient circularization method that leaves a scar sequence in the product. **c** RNAs have been designed to enable the effective circularization of scarless circRNAs. **d** The sequence of interest has been permuted; thus, it resembles exonic sequences in the T4 *td* gene. The yellow and orange boxes indicate natural introns and ribozymes, respectively. Sequences that bring the splice sites together are shown as cyan boxes. Genes of interest are represented as green boxes. Promoters and SV40 terminators are shown as gray circles and red hexagons, respectively. DNA constructs are depicted with black lines, and RNA transcripts are depicted with gray lines. RNA transcription is indicated with blue arrows. FL flag, NLS nuclear localization signal, PUF PUF binding motif, DIM dimerization domain, FPG split GFP, H homology arm, E1 and E2 exonic sequences, CDS coding sequence, L ligation sequence, GOI gene of interest.

Earlier studies used naturally occurring introns from highly expressed circRNAs to ensure robust transcription and circularization of circRNAs^19,24–28^ (Fig. 1a). However, these constructs produced a mixture of RNA forms, i.e., circRNAs and linear RNAs, suggesting that the constructs must be optimized to robustly transcribe the intended linear RNAs and induce their circularization via back-splicing in vivo.

As a growing number of cis- and trans-acting factors involved in back-splicing have been identified, studies aimed at ectopically expressing circRNAs using these factors have been performed (Table 2). Researchers have simulated natural introns by inserting intronic complementary sequences (ICSs) that bring splice sites together^24,29–32^ (Fig. 1b). Qi et al. described the engineering of circRNA regulators, which combine circRNA vectors with the RNA-binding motifs of homodimerizing RNA-binding proteins (RBPs) and nuclear localization signals^33^ (Fig. 1b). Introduction of the RNA-binding domains from PUM1 paired with those from ZBTB18, HNRNPA1, and PRKAR1A resulted in expression levels similar to those of ICSs without affecting linear RNA expression.

**Table 2:** RNA-binding proteins known to affect exon circularization.

Factors	Effect	Reporter	Sample	Mechanism	Reference
ADAR1	−	Total RNA-seq	HEK293, mouse P19 EC, SH-SY5Y	Destabilizes RNA pairs via RNA editing	^59,71^
AQR	+	circmCherry	HeLa	Unknown	^32^
B52 (SRSF6)	−	circLaccase2, circPlexA	DL1	Unknown	^26^
CASC3	−	circmCherry	HeLa	Unknown	^32^
CDC40	−	circPlexA	DL1	Unknown	^73^
Cdc5	−	circPlexA	DL1	Unknown	^73^
DDX5	−	circmCherry	HeLa	Unknown	^32^
DHX9	−	Total RNA-seq	HEK293	Destabilizes RNA pairs by resolving inverted Alu pairs	^116^
EFTUD2	−	circPlexA	DL1	Unknown	^73^
ESRP1	+	circBIRC6	hESC	Directly associates with the flanking introns	^117^
Fus	+	Total RNA-seq	mESC-derived motor neurons	Directly bridges the junctions	^118^
GEMIN5	+	circmCherry	HeLa	Unknown	^32^
HNRNPA1	−	circmCherry	HeLa	Unknown	^32^
HNRNPA3	+	circmCherry	HeLa	Unknown	^32^
HNRNPH2	−	circmCherry	HeLa	Unknown	^32^
HNRNPK	+	circmCherry	HeLa	Unknown	^32^
HNRNPL	+	circmCherry	HeLa	Unknown	^32^
HNRNPL	−	Total RNA-seq	LNCaP	Directly associates with the junctions and upregulates circRNA synthesis	^72^
HNRNPM	−	Total RNA-seq	LNCaP	Binds to long introns	^119^
KHSRP	+	Total RNA-seq	K562, HepG2	Directly associates with flanking introns	^120^
LSM5	+	circmCherry	HeLa	Unknown	^118^
MBL	+	circMbl	S2, HEK293	Directly bridges the junctions	^67^
NCBP2	+	circmCherry	HeLa	Unknown	^72^
NF90/NF110	+	Total RNA-seq	HeLa	Stabilizes intronic RNA pairs	^72^
NOVA2	+	Total RNA-seq	mouse whole cortex, cortical neurons	Binds to YCAY motifs in the flanking introns	^121^
PCBP1	+	circmCherry	HeLa	Unknown	^72^
PCBP2	+	circmCherry	HeLa	Unknown	^72^
PHAX	+	circmCherry	HeLa	Unknown	^72^
Phf5a	−	circPlexA, circUex	DL1	Unknown	^73^
PPIE	+	circmCherry	HeLa	Unknown	^32^
PPP1R8	−	circmCherry	HeLa	Unknown	^32^
Prp3	−	circPlexA	DL1	Unknown	^73^
Prp6	−	circPlexA	DL1	Unknown	^73^
Prp8	−	circPlexA	DL1	Unknown	^73^
QKI	+	circSMARCA5 reporter, total RNA-seq	mesHMLE	Directly bridges the junctions	^27^
RBM20	+	Titin isoforms	Mice	Directly bridges the junctions	^122^
RBM26	−	circmCherry	HeLa	Unknown	^32^
RBM33	−	circmCherry	HeLa	Unknown	^32^
RBM38	−	circmCherry	HeLa	Unknown	^32^
RBM4B	+	circmCherry	HeLa	Unknown	^32^
SCAF1	+	circmCherry	HeLa	Unknown	^32^
SF1	+	circmCherry	HeLa	Unknown	^32^
SF2 (SRSF1)	−	circPlexA, circUex, circLaccase2	DL1	Unknown	^26,73^
SF3a1	−	circPlexA, circUex	DL1	Unknown	^73^
SF3a2	−	circPlexA, circUex	DL1	Unknown	^73^
SF3A2	−	circHomer1	Rat hippocampal neurons	Unknown	^77^
SF3a3	−	circPlexA, circUex	DL1	Unknown	^73^
SF3b1	−	circPlexA, circUex	DL1	Unknown	^73^
SF3B1	−	circHomer1	Rat hippocampal neurons	Unknown	^77^
SF3B14	+	circmCherry	HeLa	Unknown	^32^
SF3b2	−	circPlexA, circUex	DL1	Unknown	^73^
SF3b3	−	circPlexA, circUex	DL1	Unknown	^73^
SF3b4	−	circPlexA, circUex	DL1	Unknown	^73^
SF3b5	−	circPlexA, circUex	DL1	Unknown	^73^
SF3b6	−	circPlexA, circUex	DL1	Unknown	^73^
SFPQ	+	Total RNA-seq	HEK293T, HepG2	Binds to flanking introns	^123^
Slu7	−	circPlexA	DL1	Unknown	^73^
SLU7	+	circmCherry	HeLa	Unknown	^32^
snRNP-U1-70K	−	circPlexA	DL1	Unknown	^73^
snRNP-U1-C	−	circPlexA	DL1	Unknown	^73^
SNRPA	+	circmCherry	HeLa	Unknown	^32^
SNRPC	+	circmCherry	HeLa	Unknown	^32^
SRp54 (SRSF11)	−	circLaccase2, circPlexA	DL1	Unknown	^26^
TARDBP	+	circmCherry	HeLa	Unknown	^32^
U1 snRNP	−	circEFM5, circHMRA1	*S. cerevisiae*	Unknown	^76^
U4/U5/U6 tri-snRNP recruitment	−	Total RNA-seq	Rat hippocampal neurons	Long and repeat-rich introns facilitate circRNA formation under spliceosome depletion	^77^

Meganck et al. sought to increase the circularization efficiency of natural introns by partially deleting ZKSCAN1 and HIPK3 introns with inverted ALU elements^19^. In a more recent study, a circPVT1 backbone was used because shorter exonic sequences did not circularize efficiently with the widely used ZKSCAN1 introns^34^. In 2021, the same group generated a series of insertions and deletions in the upstream and downstream introns of the model to investigate the effect of the distance between the ALU elements and the splice junction^35^. These experiments revealed that truncating the upstream and downstream introns to bring the ALU elements closer to the splice junction enhanced circRNA and protein expression by up to fivefold. The authors stated that systems with synthetic introns have multiple advantages compared to the Tornado system^36^, which utilizes the “Twister” ribozyme, whose splicing leaves RtcB-compatible reactive RNA ends, because the synthetic introns can be further improved, and their short lengths enable researchers to package the system into recombinant adeno-associated viral (AAV) vectors, allowing long-term gene expression in a wide range of tissues^37,38^. Controlling the amount of circRNA expressed in cells can be difficult, as transcription and circularization may be affected by endogenous factors.

### In vitro RNA circularization

Another common method for inducing RNA circularization involves the use of a permuted intron‒exon system (PIE), which comprises exonic sequences flanked by group I self-splicing introns^39^. This method enables the expression of desired circRNAs both in vitro and in vivo. Anabaena pre-tRNA introns and T4 bacteriophage *td* gene introns are widely used with some modifications^20^ (Fig. 1c). Litke et al. devised the Tornado system, which utilizes ubiquitously expressed, tRNA precursor-ligating, RtcB-compatible 5ʹ and 3ʹ ends and “Twister” ribozymes^36,40^ (Fig. 1c). Further studies have shown that the Tornado system can robustly express stable circRNAs in vivo and that these circRNAs play designated biological roles^41^. However, the caveat of the Tornado system is that ribozyme-based circularization leaves an unintended exonic sequence, called a “scar”, in the resulting circRNAs. Generating circRNAs based on group I introns inevitably leaves 80–180 nt long sequences derived from the two adjacent exons (commonly referred to as E1 and E2) in the products. These scars introduce undesired sequences into the final product, and these sequences elicit immune responses or have unexpected effects on the experimental results.

### Strategies for generating “scarless” circRNAs

To overcome the scar issue, Rausch et al. screened for possible exon‒intron pairs necessary for the self-splicing of T4 *td* introns and suggested permuting desired exonic sequences to ensure that the 5ʹ- and 3ʹ-termini resemble the E2 and E1 exonic sequences of the T4 *td* gene^42^ (Fig. 1d). The authors demonstrated that their constructs could easily produce circRNAs without T4 exon sequences in vitro. Unlike transfection of the Tornado system, transfection of the modified scarless system did not induce an immune response^21,43^. Efforts to identify methods for synthesizing scarless circRNAs are ongoing and represent an active field of research. Zuo et al. devised a novel strategy, termed Clean-PIE, that could be applied in vivo and in vitro using permuted T4 *td* introns^44^. The authors concealed the E1 and E2 sequences necessary for the splicing reaction in the ORF of their construct and optimized the variable parts of the E1 and E2 sequences. In another study by Wang et al., group II introns were used to produce scarless circRNAs in vitro^45^. In this study, the exon-binding site in the D1 domain of group II introns was modified; therefore, it could bind to the circular exon for self-splicing. This backbone is not universally applicable because the sequence of circular exons differs among genes; thus, the D1 sequence should be modified differently for different genes.

Another group adopted the group I intron of *Tetrahymena*, a trans-splicing ribozyme that enables the efficient circularization of RNAs without scars. Lee et al. concatenated the target sequence (5ʹ-NNNNNU-3ʹ) recognized by the *Tetrahymena* intron at the 3ʹ end of the gene of interest and the intron itself, allowing end-to-end self-targeting and splicing to occur^46^. The results indicated that this system could induce more robust expression of circRNAs than the PIE method, although the authors stated that self-circularization was effective only in vitro. They investigated whether this system could generate multimeric circRNAs via intermolecular splicing and concluded that this was unlikely. The authors recommended that only a single target site be present to achieve precise splicing. Similarly, Cui et al. devised a construct with a backbone and flanking antisense sequences to aid in the self-splicing of *Tetrahymena thermophila* introns^47^. The efficiency was approximately 80% both in vitro and in vivo. They synthesized circFOXO3 using this method and found that the product could be utilized to regulate various cellular phenotypes, such as proliferation, migration, and apoptosis, in prostate cancer cells.

### Generation of circRNAs with chemicals or enzymes

CircRNAs can be generated in vitro from linear precursors via reactions catalyzed by chemicals or enzymes^48^. Generally, RNA synthesis using chemicals results in the production of short oligomers (~50–70 nt). Therefore, an additional step of ligating several RNAs is required to synthesize larger molecules. Another challenge in the chemical circularization of RNA is that the concentration of the linear precursor should be low to prevent its oligomerization, which leads to low throughput. This method requires preorientation of the two reactive ends, which may be performed using a linear or hairpin helper oligonucleotide or a splint.

Several enzymes can be used for intramolecular ligation; the most commonly used are T4 DNA ligase and T4 RNA ligases 1 and 2. Specifically, T4 RNA ligase produces large amounts of homogenous and pure circRNAs^49^.

Although circularization by chemical reactions has many disadvantages, each method has its strengths and limitations, and the method should be chosen based on several characteristics of the circRNA product of interest (i.e., in vivo or in vitro production, natural or modified nucleotides, and construct size)^48^. The length of the sequence of interest could limit the choice of synthesis method owing to the difficulties in synthesizing large molecules using chemical methods and the PIE system^39^. In fact, PIE system does not work if there are long (1.1 kb) intervening regions between the splice sites^20^. Additionally, long RNAs tend to be less efficiently circularized and are more prone to nicking when magnesium ions are present during and after IVT^20^. Chemical- or enzyme-based methods produce circRNAs only in vitro, whereas methods based on ribozymes can robustly generate circRNAs both in vitro and in vivo. Therefore, ribozyme-based methods are frequently used to express endogenous RNA sequences.

## Design of messenger circRNA vectors

**Fig. 2:**
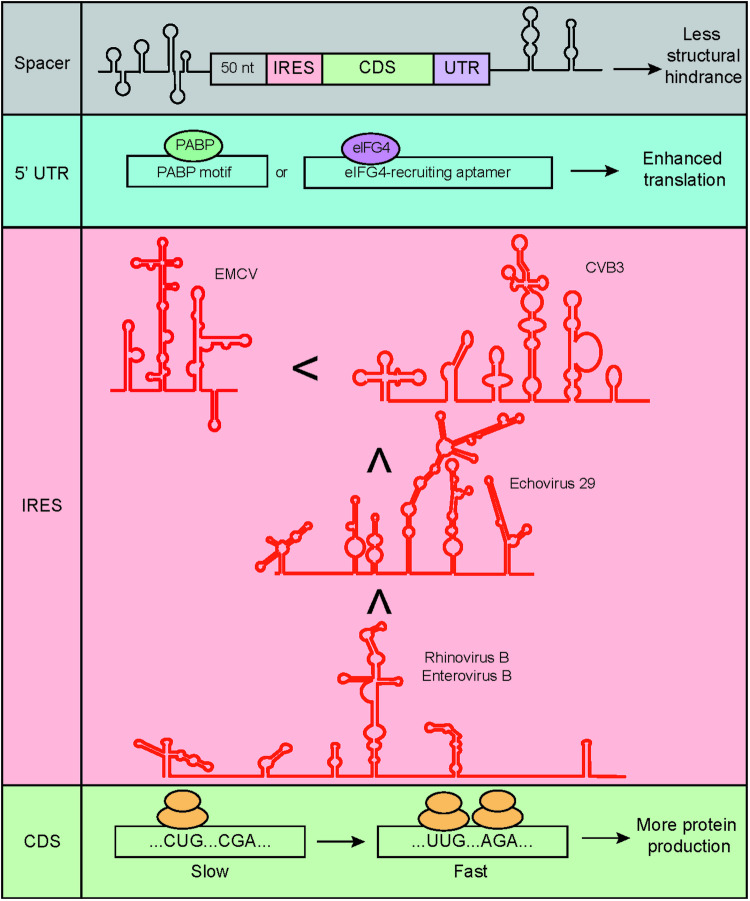
Rational design of circRNA sequences. Each factor to be considered is presented for each region of the vector of interest. Spacers were added to lessen the structural hindrance between the IRES and the gene of interest or the splice junction. Adding motifs for RBPs that enhance translation is beneficial. Several IRESs with stronger activity than the canonically used IRESs and additional codon optimization can result in faster and more abundant protein production.

Synthesizing “messenger circRNAs” that encode polypeptides requires the design of circRNA vectors that consider cis-acting factors to robustly express the desired protein (Fig. 2). The choice of the IRES, 5ʹ- and 3ʹ-untranslated regions (UTRs), and coding region can affect the translation efficiency^50^.

Vector topology is crucial for IRES-mediated translation, as the IRES is a structural element that recruits ribosomes. It is important to ensure that the sequences flanking the IRES do not interfere with IRES activity by forming complex secondary structures. In this case, the addition of spacers to separate each secondary structure can facilitate translation. One study reported that the addition of spacers to attenuate the structural hindrances caused by IRESs can improve translation^20^. A more recent study concluded that placing spacers of 50 nt in length between the splicing scar of T4 *td* introns and the IRES resulted in the most robust translation^50^. Furthermore, Liu et al. showed that RNA duplexes in circRNAs may activate degradation by PKR^51^. Thus, it is important to design the overall sequence to minimize the formation of RNA duplexes.

The IRES of encephalomyocarditis virus is the most commonly used IRES owing to its robust and nonspecific expression, which does not require many IRES trans-acting factors^19,20,35,52^. However, an extensive comparison of various IRESs revealed that an IRES from coxsackievirus B3 (CVB3) was the most efficient across several cell lines (HEK293, HeLa, A549, and Min6)^20^. Further investigation revealed that the IRES of Echovirus 29 had a stronger translation signal than the IRES of CVB3^44,53^. A later study investigated a wide range of viral IRESs to optimize circRNA translation; the authors concluded that IRESs of human rhinovirus B and enterovirus B species could drive strong translation and further elucidated that the translation efficiency of viral IRESs could be further improved by the insertion of eukaryotic translation initiation factor (eIF) G4-associated aptamers^50^. Moreover, random sequences were generated in this study to screen for IRES activity, and several sequences with strong translation-driving power were identified^50^.

UTR sequences are known to regulate multiple aspects of RNA translation, post-transcriptional regulation, and RNA stability^54^. UTRs harbor many sequences and structural elements that positively or negatively affect translation. One of the well-characterized examples is the binding site for poly(A)-binding proteins (PABPs) in the 5ʹ UTR, which aids in the binding of eIFs^55^; in addition, a highly structured 5ʹ-UTR is known to attenuate translation efficiency^56^. Including poly(A)^20^ or poly(AC)^44^ sequences in the construct improved translational strength and reduced immunogenicity^16^. Chen et al. noted that adding PABP motifs and an aptamer sequence that recruits eIF4G increased the translation of the circular reporter^50^. A few 3ʹUTRs of linear mRNAs, such as that of human β-globin, have been shown to enhance protein production^57^. Most of the 3ʹUTRs that tend to drive efficient translation of linear RNAs, except for the 3ʹUTR of human α-globin 2, do not seem to do so for circRNAs^50^.

Codon triplets are recognized by tRNAs during translation, and it has long been debated whether codon usage and the abundance of tRNAs can affect translation efficiency and speed^58^. The kinetics of translation are crucial for proper protein folding and translation elongation^59,60^; therefore, optimizing codons for the same amino acid may promote effective protein production. This field of study has not been rigorously explored; however, it was shown that eliminating unfavorable base-pairing interactions between the adjacent ends of an IRES and a coding sequence can further facilitate circRNA translation^50^.

## Delivery of synthesized circRNA or DNA constructs

During IVT-mediated circRNA synthesis, the resulting molecules must be rigorously purified. Purification by gel extraction or size-exclusion high-performance liquid chromatography is necessary because Anabaena introns or rare circular concatenations are resistant to degradation by RNase R^20^. The solid-phase DNA probe method^61^, in which a DNA probe is designed to hybridize the back-splice junction of the desired product^62^, can also be used to purify circRNAs from total RNA.

The size of the construct and the required targeting specificity can influence the choice of delivery vehicle. CircRNAs can be efficiently delivered using lipids^21^, gold nanoparticles^63^, AAV vectors^19^, lentiviral vectors, exosomes^64^, and transposons^65^. Recent advances in nanodrug delivery have suggested that nanoparticles may increase target specificity^66^. Although each method has distinct limitations and strengths, the toxicity of gold nanoparticles is under debate. Exosomes may be more biocompatible than nanoparticles but require complex manufacturing processes. For the delivery of naked circRNAs, optimization of the solvent may result in greater cellular uptake, as shown in a study by Yang et al., in which the use of Ringer’s solution resulted in the highest reported uptake at tumor sites^53^.

## Intracellular regulation of circRNA expression

**Fig. 3:**
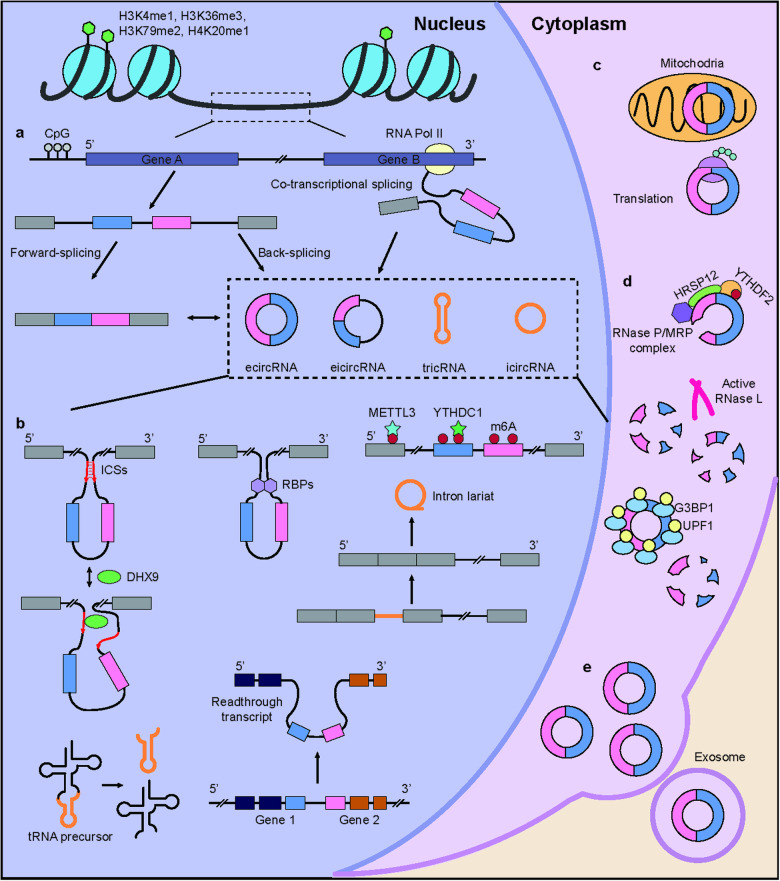
Biogenesis and regulation of circRNAs during their lifespan. **a** Epigenetic and transcriptional regulation of circRNAs. **b** Mechanisms of circRNA biogenesis. **c** CircRNA translation and subcellular localization. **d** Mechanisms of circRNA decay. **e** Cellular export of circRNAs.

Endogenous circular RNAs exhibit tightly regulated expression, are stable with a long half-life, and are resistant to RNA decay mechanisms. The level of circRNAs is affected by numerous factors in multiple steps; therefore, several factors need to be considered to achieve stable expression (Fig. 3).

### Regulation of back-splicing

Back-splicing efficiency is a combination of numerous factors at multiple levels, including chromatin states and sequence context in exons and flanking introns^25,67^ (Fig. 3a). At the epigenomic level, several histone modifications, including H3K4me1, H3K36me3, H3K79me2, and H4K20me1, affect circRNA biogenesis^68^. However, some exons are more preferentially processed by circRNAs than others, and the more back-splicing that occurs on an exon, the more exon skipping occurs during forward splicing^24,69^. However, exon skipping does not guarantee the inclusion of the exon in a circRNA; therefore, an additional level of regulation is required for exon circularization^69^. There are a few reported cases of *Schizosaccharomyces pombe* in which circRNA biogenesis occurs independently of cis or trans elements^5^, despite various cis- or trans-acting factors having been reported to facilitate or hinder circRNA production. ICSs are among the most important cis-acting elements, although their importance varies among species^10^. ICSs can be either inverted repeats^9,25^ or nonrepetitive elements^24,70^. In human fibroblasts, the vast majority (88%) of ICSs contain ALU repeats^9^.

In addition to cis-acting factors, RBPs can promote or disrupt exon circularization (Table 2). Quaking binds to flanking introns and forms a homodimer, bringing the splice sites together^27^. In contrast, A-to-I editing protein (ADAR1) can destabilize RNA pairs necessary for back-splicing via A-to-I editing^71^. However, the role of RBPs in circRNA biogenesis requires further investigation, as the effect of RBPs may vary depending on the type of circRNA and cell line. For example, HNRNPL increases the expression of the circmCherry reporter in HeLa cells^32^; however, a study of HNRNPL-knockdown LNCaP cells showed that endogenous circRNAs were downregulated rather than upregulated^72^ (Table 2). Similarly, knockdown of Slu7 resulted in circRNA enrichment in DL1 cells^73^ and depletion of circmCherry in HeLa cells^32^.

Back-splicing is performed by the spliceosome machinery and involves canonical splice sites in most cases (99%); therefore, it competes with forward splicing, although forward splicing is >100-fold more efficient^74,75^. Thus, the inhibition of forward splicing may be important for promoting back-splicing. Ablation of some core factors of the spliceosomal complex and treatment with a splicing inhibitor allows more back-splicing events to occur^73,76,77^. Despite the expected competition between forward and back-splicing, early genome-wide studies have shown that the levels of circular and linear isoforms are not fully correlated with each other^7,27,59,78,79^, although researchers have made further efforts to define the efficiency of back-splicing in terms of the circular-to-linear ratio (CLR). The CLR is the ratio of mapped sequencing reads that support back-splicing to those that support forward splicing^59^. Although the CLR is known to be <1% for most human loci, some circRNAs are robustly expressed and sometimes accumulate to levels that exceed those of their corresponding linear forms^7,59,78^. However, the precise mechanism underlying the regulation of back splicing efficiency remains to be elucidated.

### Context specificity of circRNA expression

CircRNAs are known to show expression patterns that are strongly specific for certain biological conditions and independent of those of linear isoforms, increasing the difficulty of understanding the control of circRNA expression^27,59,80,81^. A recent study using 90 human tissue transcriptomes revealed that 36–75% of alternative back-splicing events are tissue-specific^82^. Investigation of tissue-wide circRNA profiles revealed that different brain compartments, such as the olfactory bulb, prefrontal cortex, hippocampus, and cerebellum, have the greatest number of tissue-specific circRNAs^59^. By comparison, the heart, liver, and muscle have the lowest number of tissue-specific circRNAs^83^. These characteristics are not limited to endogenous circRNAs. Advances in the engineering of synthetic circRNAs have revealed similar tissue- and cell-type specificities for exogenous circRNAs. Injecting AAV vectors carrying sequences encoding the circular form of green fluorescent protein into mice resulted in different transduction rates across tissues^19^, although AAV vectors are known to broadly express encoded sequences without any tissue preference^84^.

CircRNA specificity extends beyond the tissue or cell type level to include cell-to-cell variations and distinct subcellular localization patterns (Fig. 3c). Single-cell studies have reinforced the idea that circRNA profiles vary from cell to cell^85–87^. Little is known about the subcellular localization of circRNAs; however, exonic circRNAs are localized mostly in the cytoplasm, whereas those with intronic sequences primarily remain in the nucleus^2,5,88,89^. The nuclear export of some circRNAs appears to be mediated by their length-dependent association with UAP56 or URH49, which are RNA helicases that recruit the REF adapter protein to RNAs^90^. Another study showed that YTHDC1, an m^6^A reader protein, mediates the nuclear export of circNSUN2 via m^6^A modification; this was the first report of an association between m^6^A and circRNA translocation^91^. In a more recent study, circRNA representation in the nuclear, cytoplasmic, mitochondrial, ribosomal, cytosolic, and exosomal fractions of HepG2 cells was systematically examined^92^. The results indicated that circRNAs in different compartments had different characteristics regarding length and G/C content. In neurons, some circRNAs have been shown to localize to synapses^59,78^; however, the elements that dictate this localization are unknown^34^. Several studies have examined functional mitochondrial circRNAs and revealed that their intracellular expression levels are altered under stress conditions^93–96^. Overall, these results suggested that circRNA expression is tightly regulated at different subcellular locations. Data from continued efforts to investigate the subcellular localization of circRNAs have been integrated into platforms for the visual presentation of localization information^97^.

### Regulation of the intracellular levels of circRNAs

One prominent feature that distinguishes circRNAs is their marked stability. Researchers have found that the half-lives of circRNAs are, on average, two- to fourfold longer than those of linear mRNAs and sometimes as much as 10-fold longer^98^. This difference results mainly from the absence of 5ʹ- and 3ʹ-terminal nucleotides that can be attacked by exonucleases, which block the degradation of circRNAs under normal or stressful conditions. During viral infection, RNase L is activated via an unknown mechanism and globally degrades circRNAs associated with PKR as part of the innate immune response^51^ (Fig. 3d). Park et al. identified RNase P and MRP as circRNA-degrading agents that interact with YTHDF2 and HRSP12, two proteins that recognize circRNAs with m^6^A modifications and a GGUUC motif^99^. *Drosophila* GW182, a key component of P-bodies, and its human homologs TNRC6A/TNRC6B/TNRC6C participate in circRNA decay through an AGO2-independent mechanism, and their depletion substantially increases the steady-state levels of cytoplasmic circRNAs^100^. Considering that these mechanisms function sequentially, it is likely that circRNA isoforms are subjected to decay via different mechanisms, possibly leading to the enrichment of circRNAs related to stress responses.

Under normal conditions, approximately one-third of human circRNAs are predicted to be highly structured, and their degradation is globally regulated by UPF1 and G3BP1 via structure-mediated RNA decay (SRD)^101^. G3BP1 selectively binds to highly structured circRNAs and is a determining factor in SRD. SRD targets appear to be preferentially excluded from stress granules, where UPF1 and G3BP1 localize after stress-inducing treatment. Taken together, these results indicate that circRNA decay mechanisms vary between normal and stressful conditions. None of the factors mentioned above exclusively target circRNAs; thus, it is likely that there are additional unknown pathways responsible for the regulation of circRNA steady-state levels^102^.

Studies using human cell lines have suggested that circRNAs can be actively exported^103^ (Fig. 3e). Several reports have shown that circRNAs are enriched in extracellular vesicles^103,104^, in the circulation and urine^105^, and in exosomes secreted by various cell lines^106,107^. Additionally, circRNAs with a 5ʹ-GMWGVWGRAG-3ʹ motif were found to be selectively packaged into exosomes^92^. However, the exact mechanism of circRNA secretion and its effect on donor and recipient cells remain unknown^108^.

## Immunogenicity of exogenous circRNAs

The immunogenicity of engineered circRNAs remains controversial. Chen et al. showed that transfection of circRNAs using a PIE system containing the T4 *td* gene intron triggered the expression of several immune genes, whereas transfection of a circRNA generated with the ZKSCAN1 intron did not^43^. Subsequently, they observed that m^6^A modification could act as a molecular marker for “self” circRNAs^109^. Another study using the Anabaena intron reported that the resulting circRNA did not elicit an immune response^20^. This was later challenged by a more recent study, which concluded that circRNAs produced by group I introns are immunogenic, possibly due to the intron “scars” that remain in the final product^110^. This inconsistency could be a result of differences in the methods used to test immunogenicity or the type of linear RNA used for comparison since the above studies all used distinct methods to evaluate the immunogenicity of a circRNA^111^. However, the immunogenicity of vector-carrying circRNAs has not been discussed in detail. To date, the transduction of circRNAs via AAV vectors or lentiviruses has shown negligible immune activation ability.

## Application of engineered circRNAs

**Fig. 4:**
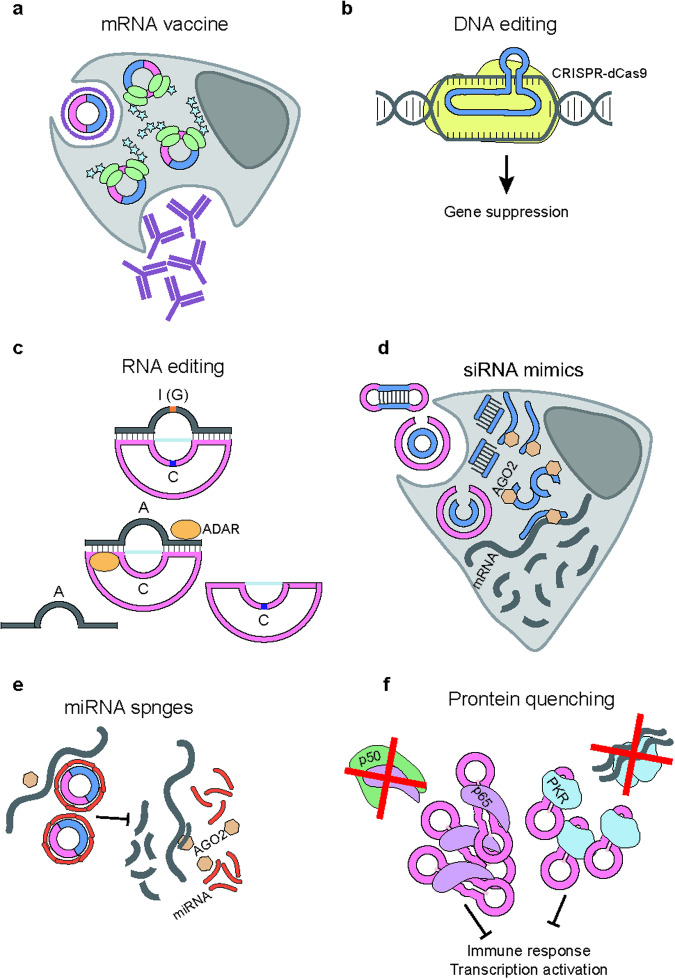
Application of circRNAs. CircRNAs can be used as mRNA vaccines (**a**) and guide RNAs for DNA and RNA editing (**b**, **c**). Additionally, circRNAs function as miRNA sponges or siRNA mimics (**d**, **e**) and can be used to isolate proteins (**f**).

Recently, several studies have used circRNA technology to investigate or control cellular processes and immune responses (Fig. 4) ^21,36,43,51,109^. Circular mRNA vaccines showed efficient protection against SARS-CoV-2 infection (Fig. 4a)^15,22^. Furthermore, circRNAs aid in reducing the effective vector dose for gene therapy applications because the expression levels of their protein products are likely to increase over time^19^.

CircRNAs have also been used in DNA editing and RNA regulation. Two groups used ADAR with a circular guide RNA for in vivo and in vitro RNA editing (Fig. 4b, c)^112,113^. Several siRNA mimics^114^, RNA dumbbells^115^, and aptamers^36^ have been shown to perform robustly, with improved stability in the circular form (Fig. 4d–f).

## Conclusion

CircRNAs have demonstrated potential as molecules for next-generation vaccines and therapeutics. Herein, we presented several options that could be chosen and aspects that could be considered when developing a platform for circRNAs. A rational sequence design that guarantees the maximal cellular level of a circRNA or a desired protein is of pivotal importance. One could also adopt an adequate delivery method and enhance cellular uptake by optimizing the solution. CircRNAs can be expressed differently in different tissues and cell types, and efforts should be made to minimize the immunogenicity of circRNAs.

Our aim was to show how the process for exogenous circRNA synthesis can be modified to be more efficient and suitable for circRNA synthesis. By considering each step of the application of a circRNA, we believe that one can accomplish the desired results with maximum potential. Ultimately, these steps will become standard procedures for the industrial synthesis of circRNAs.
